# The complete chloroplast genome of *Cymbidium erythraeum* (Orchidaceae)

**DOI:** 10.1080/23802359.2019.1638322

**Published:** 2019-07-16

**Authors:** Jie Huang, Gui-Zhen Chen, Ting-Zhang Li, Zhi-Cong Huang, Wen-Hui Rao, Jian-Bing Chen

**Affiliations:** a Key Laboratory of National Forestry and Grassland Administration for Orchid Conservation and Utilization, Shenzhen, China;; b Shenzhen Key Laboratory for Orchid Conservation and Utilization, The National Orchid Conservation Centre of China and The Orchid Conservation and Research Centre of Shenzhen, Shenzhen, China

**Keywords:** *Cymbidium erythraeum*, chloroplast genome, Orchidaceae

## Abstract

*Cymbidium erythraeum* Lindl. is an endangered species of Orchidaceae and distributed in China and Bhutan, India, Myanmar, Nepal, and Vietnam. Here, we report the complete chloroplast (cp) genome sequence and the cp genome features of *C. erythraeum.* The complete chloroplast (cp) genome sequence of *C. erythraeum* is 156,327 bp in length and including one large single-copy region (LSC, 85,404 bp), one small single-copy region (SSC, 20,021 bp), and two inverted repeat regions (IRs, 25,426 bp). The cp genome encoded 136 genes, of which 107 were unique genes (80 protein-coding genes, 23 tRNAs, and four rRNAs). The phylogenetic relationships show that *C. erythraeum* is closely related to other species in the genus *Cymbidium* and is sister with *C. tracyanum*.

The genus *Cymbidium* was established by Swartz (1799, p. 6), and now approximately 80 species are recognized within the genus (Liu et al. 2006; Chen et al. 2009). *Cymbidium* belongs to the subtribe Cymbidiinae (Orchidaceae), ranging from tropical and subtropical Asia south to Papua New Guinea and Australia (Chen et al. 2009; Pridgeon et al. 2009). China is the distribution centre of *Cymbidium* with over 50 species and 19 of them endemic (Liu et al. 2006; Chen et al. 2009; Lan et al. 2018; Chen et al. 2019; Zhang et al. 2019). *Cymbidium* orchids are the best known and most widely grown of all orchids in worldwide horticulture because of its aesthetic appeal and ideal characteristics as a house plant.

The genomic data has been much helpful in identification and genotyping of *Cymbidium* than the previous studies (Zhang et al. 2002; Berg 2002; Yukawa and Stern 2002). In this study, the complete chloroplast genome sequence of *C. erythraeum* was assembled.

Leaf samples of *C. erythraeum* were obtained from The Orchid Conservation and Research Centre of Shenzhen, and specimens were deposited in the National Orchid Conservation Center herbarium (NOCC; specimen code Z.J.Liu 2900). Total genomic DNA was extracted from fresh material using the modified CTAB procedure of Doyle and Doyle (1987). Sequenced on Illumina Hiseq 2500 platform (San Diego, CA). Genome sequences were screened out and assembled with MITObim v1.8 (Hahn et al. 2013), which resulted in a complete circular sequence of 156,520 bp in length. The cp-genome was annotated with CpGAVAS (Liu et al. 2012).

The cp genome sequence of *C. erythraeum* (GenBank accession MK7820373) is 156,327 bp length and presented a typical quadripartite structure including one large single-copy region (LSC, 85,404 bp), one small single-copy region (SSC, 20,021 bp), and two inverted repeat regions (IRs, 25,426 bp). The cp genome encoded 136 genes, of which 107 were unique genes (80 protein-coding genes, 23 tRNAs, and four rRNAs). To confirm the phylogenetic position of *C. erythraeum*, a molecular phylogenetic tree was constructed based on the maximum-likelihood (ML) methods with twelve species from *Cymbidium.* The ML analysis was performed using the CIPRES Science Gateway web server (RAxML-HPC2 on XSEDE 8.2.10) with 1000 bootstrap replicates and settings as described by Stamatakis et al. (2008). The results showed that *C. erythraeum* is mostly related taxa with *C. tracyanum* which forms a sister group to the *Cymbidium* (Figure 1). This newly reported chloroplast genome provides a good foundation for the identification and genotyping of *Cymbidium* species.

**Figure 1.**
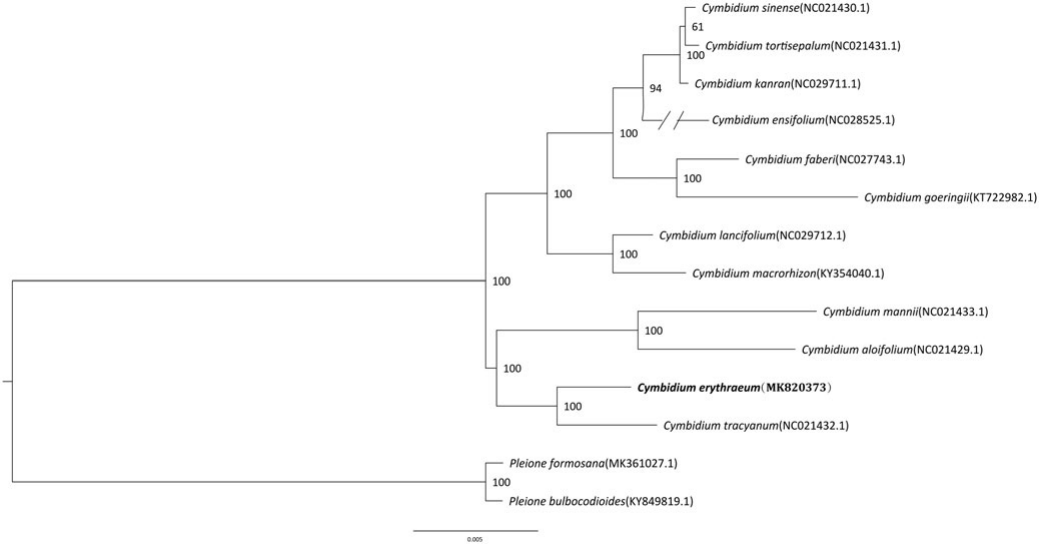
Phylogenetic position of *Cymbidium erythraeum* inferred by maximum-likelihood (ML) of complete cp genome. The bootstrap values are shown next to the nodes.
